# The importance of growth kinetic analysis in determining bacterial susceptibility against antibiotics and silver nanoparticles

**DOI:** 10.3389/fmicb.2014.00544

**Published:** 2014-11-10

**Authors:** Karsten Theophel, Veronika J. Schacht, Michael Schlüter, Sylvia Schnell, Catalina-Suzana Stingu, Reiner Schaumann, Michael Bunge

**Affiliations:** ^1^Institute of Applied Microbiology, Research Center for BioSystems, Land Use, and Nutrition, Justus Liebig University of GiessenGiessen, Germany; ^2^Institute for Medical Microbiology and Epidemiology of Infectious Diseases, University of LeipzigLeipzig, Germany

**Keywords:** *Enterococcus*, antibiotic susceptibility testing, growth dynamics, subinhibitory, hormesis, antimicrobial nanoparticles, silver

## Abstract

Routine antibiotics susceptibility testing still relies on standardized cultivation-based analyses, including measurement of inhibition zones in conventional agar diffusion tests and endpoint turbidity-based measurements. Here, we demonstrate that common off-line monitoring and endpoint determination after 18–24 h could be insufficient for reliable growth-dependent evaluation of antibiotic susceptibility. Different minimal inhibitory concentrations were obtained in 20- and 48 h microdilution plate tests using an *Enterococcus faecium* clinical isolate (strain UKI-MB07) as a model organism. Hence, we used an on-line kinetic assay for simultaneous cultivation and time-resolved growth analysis in a 96-well format instead of off-line susceptibility testing. Growth of the *Enterococcus* test organism was delayed up to 30 h in the presence of 0.25 μg mL^-1^ of vancomycin and 8 μg mL^-1^ of fosfomycin, after which pronounced growth was observed. Despite the delayed onset of growth, treatment with fosfomycin, daptomycin, fusidic acid, cefoxitin, or gentamicin resulted in higher maximum growth rates and/or higher final optical density values compared with antibiotic-free controls, indicating that growth stimulation and hormetic effects may occur with extended exposure to sublethal antibiotic concentrations. Whereas neither maximum growth rate nor final cell density correlated with antibiotic concentration, the lag phase duration for some antibiotics was a more meaningful indicator of dose-dependent growth inhibition. Our results also reveal that non-temporal growth profiles are only of limited value for cultivation-based antimicrobial silver nanoparticle susceptibility testing. The exposure to Ag(0) nanoparticles led to plasma membrane damage in a concentration-dependent manner and induced oxidative stress in *Enterococcus faecium* UKI-MB07, as shown by intracellular ROS accumulation.

## INTRODUCTION

Antibiotics are commonly used for the medical treatment of both human and animal diseases, and they are also used for prophylaxis in veterinary medicine and promotion of animal growth in intensive livestock farming (Barton, 2000; Sarmah et al., 2006; Singer et al., 2007; Marshall and Levy, 2011). The continued widespread overuse and misuse of antibiotics and the release of increasing amounts of antibiotics into the environment is cause for concern. Inappropriate wastewater treatment technologies combined with the application of antibiotic-containing liquid manure onto agricultural fields and their direct release into surface waters has resulted in extensive and continuous release of antibiotics into the environment (Sarmah et al., 2006; Kümmerer, 2009a). This is thought to be an important triggering factor in the emergence and spread of antibiotic resistance, which has in turn resulted in an increased prevalence of infections with antibiotic-resistant bacteria (Kümmerer, 2009b; Martinez, 2009a,b; Marshall and Levy, 2011; Rizzo et al., 2013). Moreover, resistant bacteria serve as genetic pools for the further spread of resistance genes among other microorganisms (Zhang et al., 2009; Rizzo et al., 2013).

Gram-positive enterococci are an important source of nosocomial infections, with high and partly increasing rates of incidence and mortality (European Antimicrobial Resistance Surveillance System [EARSS] Annual Report, 2008; Fisher and Phillips, 2009; Arias and Murray, 2012; Victorian Advisory Committee on Infection Control [VACIC], 2012). In addition to other severe infections, opportunistic *Enterococcus* pathogens can cause urinary tract infections, endocarditis, bacteremia, and sepsis, and their emergence in recent years corresponds to an increase in glycopeptide and high-level aminoglycoside resistance (HLAR; European Antimicrobial Resistance Surveillance System [EARSS] Annual Report, 2008; Victorian Advisory Committee on Infection Control [VACIC], 2012). Some *Enterococcus* spp. show the highest prevalence of clinical vancomycin resistance, due to the acquisition of antibiotic resistance genes, most likely from sources within the hospital or via routes involving intensive poultry farming (Uttley et al., 1988; Klare et al., 2003; Lester et al., 2006; Arias and Murray, 2012).

Evaluating the efficacy of antibiotics may result in potent treatments for infectious diseases. Because nosocomial infections caused by antibiotic-resistant bacteria frequently result in serious complications, such as sepsis, pneumonia, and even death, proven treatments with antibiotics that are effective against these organisms could help reduce their toll. Due to the exorbitant costs of intensive care treatment of nosocomial infections, targeted and more responsible use of antibiotics will also decrease related follow-up costs for the healthcare system and social framework (Eber et al., 2010).

Reliable testing and monitoring of antibiotic efficacy is necessary as a first step toward development of a reasonable and sustainable plan for the use of antibiotics. Whereas molecular methods are increasingly used in research and development, routine testing still requires cultivation-dependent analyses, including the use of conventional susceptibility tests (e.g., off-line determination of microbial growth in the presence of antibiotics, such as measurement of inhibition zones in agar diffusion tests, turbidity-based measurements, and the counting of colony-forming units after serial dilution; Jorgensen and Ferraro, 2009). Applied methods, including standardized techniques (e.g., those recommended by the Clinical and Laboratory Standards Institute [CLSI], 2013 or by The European Committee on Antimicrobial Susceptibility Testing [EUCAST], 2014), are based on either diffusion (e.g., Kirby-Bauer and Stokes tests), dilution [determination of the minimal inhibitory concentration (MIC)] in serial dilutions), or diffusion and dilution (e.g., E-test method; Jorgensen and Ferraro, 2009). A common feature of all of the methods described above is that they rely on off-line measurement (i.e., endpoint or regular-interval growth determination); however, these methods are not intended for monitoring microbial growth kinetics with high temporal resolution in the presence of antibiotics.

Antibiotics exert specific effects on growing microorganisms. For example, an antibiotic may impair cell wall synthesis, alter the chromosomal topology by targeting DNA gyrase, and interfere with synthesis of DNA, RNA, and proteins (Kohanski et al., 2010). This suggests that in addition to dynamic exposure scenarios, target organisms exhibit dissimilar susceptibility to different antibiotics at different growth stages, resulting in complex and antibiotic-specific dynamic growth profiles. But surprisingly, few previous studies have addressed antibiotic-induced dynamic changes in microbial growth patterns. Bacteria have developed a diverse array of strategies to counter antibiotic toxicity, including reducing the amount of antibiotic taken up, enzymatic inactivation of antibiotics, modification of the molecular target to reduce binding, and upregulation of cellular repair mechanisms (Martinez et al., 2009; Nikaido, 2009). Antibiotic transport, initial interaction with cellular components, exertion of biocidal effects, and the development of microbial resistance mechanisms are all time- and growth-stage–dependent and therefore affect growth dynamics. In light of these factors, all of which underscore the need for time-based screening, it is remarkable that none of the existing growth-dependent antibiotic susceptibility tests has been adapted to determine microbial growth kinetics with a high degree of temporal resolution.

Our aim was to overcome the limited applicability of existing methods for studying the dynamic effects of antibiotics on microbial growth kinetics by modifying a common microdilution assay to allow simultaneous cultivation and online analysis of growth inhibition with high temporal resolution.

## MATERIALS AND METHODS

### CULTIVATION AND GROWTH ANALYSIS FOR TIME-RESOLVED SUSCEPTIBILITY TESTING

Cultivation of an *Enterococcus faecium* clinical isolate (strain UKI-MB07, isolated from material at the Innsbruck Medical University) was performed using standard MICRONAUT-S MRSA/IFSG GP4 microdilution plates (*n* = 3) for susceptibility testing of bacteria (Merlin Diagnostika GmbH, Bornheim-Hersel, Germany). The plates were closed with sterile standard-profile lids without condensation rings (polystyrene lids, art. #656161, Greiner Bio-One, Frickenhausen, Germany). Each plate contained the following antibiotics (multiple concentration range, in μg mL^-1^): ampicillin (0.5–16), cefotaxim (2–8), cefoxitin (2–16), ciprofloxacin (1–2), clindamycin (1), cotrimoxazol (16–64), daptomycin (0.5–4), doxycyclin (1–4), erythromycin (1–4), erythromycin/clindamycin (4/0.5), fosfomycin (8–64), fusidic acid (1–4), gentamicin (0.5–8/500), linezolid (1–8), moxifloxacin (0.25–2), mupirocin (4–8), oxacillin (0.25–32), penicillin G (0.125–16), rifampicin (1–8), streptomycin (1000), synercid (0.5–4), teicoplanin (0.125–16), tigecycline (0.125–1), and vancomycin (0.25–32).

The standardized inoculum for the test plates was prepared by mixing bacteria (pre-cultured for 16 h at 36 ± 0.5°C) with sterile 0.9 % NaCl solution to adjust the turbidity to that of McFarland standard No. 0.5. A 100 μL volume of the resulting suspension was pipetted into 11 mL of Mueller–Hinton II broth (Merlin Diagnostika GmbH, Bornheim-Hersel, Germany) and the mixture was homogenized, after which 100 μL was transferred into each well of the test plates using a multichannel pipette. The plates were incubated at 36 ± 0.3°C and analyzed over a period of 48 h with a 30 min temperature equilibration period before data acquisition was started. The optical density (OD) at 660 nm was determined for each well using a Tecan Infinite M200 multimode microplate reader equipped with monochromator optics (Tecan Group Ltd., Männedorf, Switzerland). During incubation, orbital shaking conditions were selected (4 mm amplitude and 15 s shaking cycles), and measurements were taken every 15 min using the multiple-reads-per-well mode (filled-circle alignment, 3 × 3 spots, five reads per well, border 2000 μm). Based on the resulting data, MICs were determined and in part interpreted according to the current CLSI Performance Standards for Antimicrobial Susceptibility Testing (Clinical and Laboratory Standards Institute [CLSI], 2012, 2013). Growth analysis was accompanied by controls cultured in the absence of antibiotics to obtain reference curves, sterile media controls with and without antibiotics, and reference strains for quality control testing (Clinical and Laboratory Standards Institute [CLSI], 2013).

The same procedure was used to examine the effect of antibiotic/silver nanoparticle combinations on growth of the test isolate, except that 100 μL of the McFarland 0.5-adjusted cell suspension was mixed with 11 mL of Mueller-Hinton Broth to which Ag(0) nanoparticles (AgPure W10, primary particle size distribution D90 < 15 nm, ras Materials, Regensburg, Germany) in aqueous buffer were added from a 5 mg mL^-1^ Ag(0) stock solution to give a final Ag(0) nanoparticle concentration of 50 μg mL^-1^. Sterile media controls with or without Ag(0) nanoparticles were also analyzed at each sampling point to determine the background signals. All test data were normalized by subtracting the background. The stability of the nanoparticle suspensions was monitored using both light and electron microscopy as well as nanoparticle tracking analysis (data not shown). For scanning electron microscopy and elemental analysis (SEM-EDX), a Phenom pro X system was used (Phenom-World, Eindhoven, The Netherlands), which was equipped with a CeB_6_ electron source, a 4-segment backscattered electron detector, a silicon drift detector for EDX analysis, and Phenom Pro Suite software. SEM-EDX analyses were acquired from *Enterococcus* sp. strain UKI-MB07 cultures grown for 36 h at 37°C in Mueller-Hinton Broth spiked with Ag(0) nanoparticles [0.006% (w/v)]. Samples were applied on silicon carriers, dried at room temperature, and analyzed at 5 kV.

### FURTHER SUSCEPTIBILITY TESTING

The *in vitro* susceptibility to antimicrobial agents of the tested *E. faecium* strain UKI-MB07 and two reference strains *E. faecalis* ATCC 29212 and *E. faecium* ATCC 700221 was further tested by determining MIC-values employing the broth microdilution technique according to ISO 20776-1 (2006) as well as Etest (Liofilchem s.r.l., Roseto degli Abruzzi, Italy) according to the EUCAST international guidelines (The European Committee on Antimicrobial Susceptibility Testing [EUCAST], 2014). The *in vitro* susceptibility results of the two reference strains were in the range of published data (European Committee for Antimicrobial Susceptibility Testing [EUCAST] of the European Society of Clinical Microbiology and Infectious Diseases [ESCMID], 2003; ISO 20776-1, 2006).

### PCR AMPLIFICATION, ANALYSIS OF 16S rDNA SEQUENCES AND FURTHER STRAIN IDENTIFICATION

Material from single colonies was utilized for DNA extraction (UltraClean^®^ Microbial DNA Isolation Kit, MO BIO Laboratories, Inc., Carlsbad, CA, USA). Isolated DNA was used as the template for almost complete 16S rDNA amplification using the universal primer pair 27F (5′-GAGTTTGATCMTGGCTCAG-3′) and 1492R (5′-ACGGYTACCTTGTTACGACTT-3′; Lane, 1991) according to established PCR protocols. Purified amplicons were sequenced by LGC Genomics GmbH (Berlin, Germany). Sequences were analyzed using MEGA software, version 5.0 (Tamura et al., 2011), and closest relatives were identified using the EMBL-EBI Fasta33 and NCBI BLAST programs.

The strain was also identified using the Rapid ID 32 Strep system (bioMérieux SA, Marcy l’Etoile, France) as well as the VITEK-MS (bioMérieux SA, Marcy l’Etoile, France). The VITEK-MS uses a direct colony method and was operated on the V2.0 Knowledge Base for clinical use. Both methods were performed according to the recommendations of the manufacturer.

### ASSESSMENT OF CELL MEMBRANE INTEGRITY

The effects of Ag(0) nanoparticles on cell membrane integrity were assessed by using the LIVE/DEAD *Bac*Light Bacterial Viability Kit (Molecular Probes, Eugene, OR; Thermo Fisher Scientific, Waltham, MA, USA), containing SYTO 9 and propidium iodide to differentiate between live bacteria with intact cell membranes from dead bacteria with damaged cell membranes. The Live/Dead *Bac*Light Bacterial Viability Kit was applied according to the manufacturer’s instructions, with the exception of using sterile 0.9 % NaCl solution and centrifugation for 30 min at 3,345 × *g* for washing steps. Duplicate aliquots (5 μL) of each sample were spotted onto wells of Teflon-coated diagnostic slides (Menzel, Braunschweig, Germany) and a Zeiss Axioplan2 epifluorescence microscope (HBO 100W) was used with different objectives (Plan-Apochromat 63 × /1.40 Oil, Plan-Neofluar 100 × /1.30 Oil) to examine cells after exposure to 0.002 or 0.006% Ag(0) nanoparticles (AgPure W10, primary particle size distribution D90 < 15 nm, ras Materials, Regensburg, Germany) compared to Ag(0)-free controls. Images were acquired with a Zeiss AxioCam MRc CCD camera, in both bright field and fluorescence mode (Zeiss filter set 15 and filter set Sp. Green HC mFISH, modified with BrightLine HC 515/LP instead of HC 494/20, AHF Analysentechnik AG, Tübingen, Germany), and processed using Zeiss AxioVision Rel. 4.8 software.

### DETECTION OF INTRACELLULAR REACTIVE OXYGEN SPECIES

The accumulation of intracellular ROS was assessed with Image-iT^®^ LIVE Green Reactive Oxygen Species (ROS) Detection Kit (Molecular Probes, Eugene, OR; Thermo Fisher Scientific, Waltham, MA, USA) according to the manufacturer’s instructions. The assay is based on carboxy-H_2_DCFDA (5-and 6-)carboxy-2′,7′-dichlorodihydrofluorescein diacetate) as marker for ROS in live cells, *tert*-butyl hydroperoxide (TBHP), a common inducer of ROS production as positive control, and Hoechst 33342 as cell-permeant nucleic acid stain. Samples derived from 0.006% Ag(0) nanoparticle treatments were checked using an epifluorescence microscope (in comparison to Ag(0)-free control incubations and positive controls incubated for 24 h in the presence of 13.3 % Manuka honey, MGO400, Manuka Health, New Zealand), and images were acquired with a digital camera as described above, but additionally using the filter set BrightLine HC 360/12 (AHF Analysentechnik AG, Tübingen, Germany) for detection of Hoechst 33342 signals.

## RESULTS

### EXPERIMENTAL DESIGN FOR ON-LINE SUSCEPTIBILITY TESTING: GENERAL PROPERTIES AND POTENTIAL

The spectrum of antibiotics provided in the microdilution plates allows for the specific detection of clinically relevant antibiotic resistance in Gram-positive opportunistic pathogens. For instance, the plates contain antibiotics that are known for their intrinsic resistance in Gram-positive bacteria (e.g., fosfomycin and fusidic acid for some staphylococci; cephalosporins and aminoglycosides for enterococci), and allow for susceptibility testing of “novel” antibiotics such as tigecycline and daptomycin as well as differentiation between *E. faecium* and *E. faecalis* via determination of synercid susceptibility. The microdilution plates also allow for the detection of vancomycin-resistance phenotypes. Potential synergistic effects between ampicillin, penicillin, or vancomycin and aminoglycosides were estimated by a HLAR screening test using gentamicin and streptomycin. Characteristics of an HLAR-strain were not detected for the *Enterococcus* isolate examined here.

Although the microtiter plate format is routinely used for endpoint growth determination and susceptibility testing (Häussler et al., 2003; Jorgensen and Ferraro, 2009), kinetic measurements are rarely used for susceptibility testing. We therefore deployed a technique for simultaneous cultivation and determination of the effects of antibiotics on microbial growth that employs automated turbidity measurements using a monochromator-based microplate spectrophotometer. To examine the dynamic effects of antibiotics over time and their impact on microbial growth kinetics, our analyses were separated by 15 min intervals. However, the interval can be easily increased or decreased depending on the desired level of temporal resolution.

### EFFECT OF VARIOUS ANTIBIOTICS ON *ENTEROCOCCUS* GROWTH DYNAMICS

**FIGURE 1:**
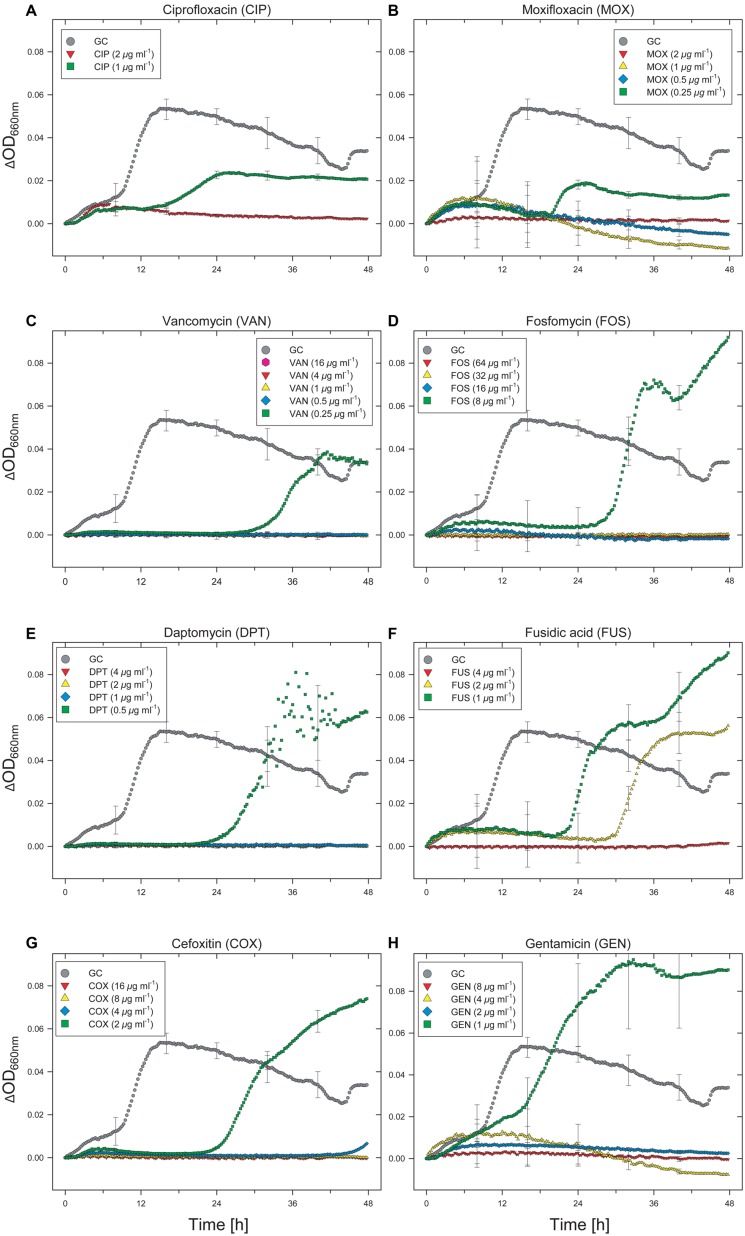
**Effect of selected antibiotics on the growth dynamics of *Enterococcus faecium* strain UKI-MB07. Note the extended lag phase and partial growth promotion at low concentrations for some antibiotics.** This hormetic effect was associated with higher maximum specific growth rates and increased OD values. Each data point (time resolution over 48 h: 15 min) represents mean values of triplicate cultivations, normalized with data from identical incubations in the absence of bacterial cells (sterile controls). Hence, each curve represents 576 single data points and kinetics from about 21,900 single data points are shown in this figure. For visual clarity, standard deviations (error bars) are only presented for selected time points.

The effects of various antibiotics on the growth dynamics of the *Enterococcus* clinical isolate were monitored using an automated 96-well microtiter plate assay that allowed simultaneous cultivation and on-line analysis of bacterial growth. By examining growth over time using the automated method, different effective exposure times and concentration-dependent effects on the growth dynamics of the *Enterococcus* clinical isolate became evident. Despite the fact that categorization of data after 48 h of incubation remains “theoretical” since we did not follow the guideline incubation time for utilizing MIC interpretative standards for *Enterococcus* spp. (dilution methods: 16 to 20 h, 24 h for vancomycin; Clinical and Laboratory Standards Institute [CLSI], 2012, 2013), for antibiotics tested at two concentrations, the *Enterococcus* isolate was sensitive to cefotaxim (2/8), doxycyclin (1/4), cotrimoxazol (16/64), mupirocin (4/8), and erythromycin (1/4) in both the 20- and 48 h experiments. According to the current breakpoint interpretation (Clinical and Laboratory Standards Institute [CLSI], 2013), these data indicate that the *Enterococcus* test isolate can be categorized as susceptible (S) to doxycyclin and as either (S) or of intermediate susceptibility (I) to erythromycin (“theoretical” categorization of 48-h data sets). Ciprofloxacin showed intermediate performance (growth at 1 μg mL^-1^ and no growth at 2 μg mL^-1^) in both the 20- and 48-h experiments; however, growth in the presence of 1 μg mL^-1^ of ciprofloxacin did not begin until about 15 h of incubation (**Figure 1A**).

A total of 15 antibiotics were tested at more than two concentrations. Only considering those seven antibiotics that were tested at more than two concentrations and showed limited activity against the *Enterococcus* isolate at the lower concentrations (moxifloxacin, vancomycin, fosfomycin, daptomycin, fusidic acid, cefoxitin, and gentamicin), six of them showed different MIC values by comparison of the 20 and 48-h data sets (**Table 1**). All of these six antibiotics showed concentration-dependent effects on basal microbial growth dynamics (**Figures 1B–H**).

**Table 1:** The minimal inhibitory concentration (MIC) of selected antibiotics against an *Enterococcus* sp. clinical isolate determined after 20 and 48 h of exposure.

Antibiotic	MIC [μg ml^-^^1^] 20 h	MIC [μg ml^-^^1^] 48 h
Ampicillin	≤0.5	≤0.5
Cefoxitin	≤2	8
Daptomycin	≤0.5	1
Fosfomycin	≤8	16
Fusidic acid	≤1	4
Gentamicin	2	2
Linezolid	≤1	≤1
Moxifloxacin	≤0.25	0.5
Oxacillin	≤0.25	≤0.25
Penicillin G	≤0.125	≤0.125
Rifampicin	≤1	≤ 1
Synercid	≤0.5	≤0.5
Teicoplanin	≤0.125	≤0.125
Tigecycline	≤0.125	≤0.125
Vancomycin	≤0.25	0.5

*Due to the limited clinical applicability of some antibiotics [e.g., restricted efficacy of cephalosporins for *Enterococcus* infections, intrinsic resistance to fusidic acid, Clinical and Laboratory Standards Institute [CLSI], 2013)] and because we did not follow the guideline incubation time for utilizing MIC interpretative standards for *Enterococcus* spp. in case of 48 h of incubation, MIC values are reported without an accompanying S, I, or R categorization*.

After a lag phase of about 9 h, control cultures without antibiotics showed a sharp increase in OD, followed by a slow decrease over the remainder of the incubation period (**Figure 1**). Extended lag phases were observed in cultures containing fluoroquinolones (ciprofloxacin, moxifloxacin) at concentrations of 1 μg mL^-1^ (ciprofloxacin) and 0.25 μg mL^-1^ (moxifloxacin; **Figures 1A,B**). For ciprofloxacin, the duration of the lag phase was 17 h (**Figure 1A**). Despite a lag phase of >20 h, cells exposed to moxifloxacin began to grow exponentially at a rate comparable to that of the antibiotic-free control (**Figure 1B**). However, for cultures exposed to both fluoroquinolones, the final OD values did not reach that of the controls.

An even longer lag phase, with a maximum duration of up to 30 h, was observed with cultures exposed to vancomycin, fosfomycin, daptomycin, fusidic acid, and cefoxitin (**Figures 1C–G**). If the recommended cultivation duration of 16–20 h (24 h for vancomycin; Clinical and Laboratory Standards Institute [CLSI], 2012) had been used, no growth would have been detected in any of the cultures containing these antibiotics. Compared with the antibiotic-free control, cells exposed to fosfomycin, daptomycin, fusidic acid, cefoxitin, and gentamicin grew at even higher rates during the logarithmic phase and reached higher final OD values. Despite the fact that some antibiotics have limited clinical efficacy in treating *Enterococcus* infections (e.g., restricted clinical efficacy of cephalosporins for *Enterococcus* infections, intrinsic resistance for fusidic acid), these results suggest that at low concentrations these antibiotics actually stimulate the growth of this organism (**Figures 1D–H**). For instance, the maximum specific growth rate in the presence of 8 μg mL^-1^ of fosfomycin was considerably higher than the control lacking antibiotic. The OD value of the fosfomycin-exposed culture exceeded that of the control after 31 h and reached a maximum value of more than twice that of the control after 48 h. Under more severe fosfomycin-induced stress, however, the *Enterococcus* isolate was unable to grow. As for all other tested antibiotics, higher concentrations completely and irreversibly inhibited growth of the *Enterococcus* isolate (**Figures 1D–H**).

A more complex concentration-dependent effect was observed in the case of fusidic acid, for which growth was observed at concentrations up to 2 μg mL^-1^ (**Figure 1F**). Whereas a lag phase of about 22 h was observed in the presence of 1 μg mL^-1^ of fusidic acid, with 2 μg mL^-1^ growth did not begin until about 30 h. The duration of the lag phase in cultures exposed to fusidic acid in the range 0–4 μg mL^-1^ was concentration-dependent (**Figure 1F**). Despite the delayed onset of growth, similar maximum specific growth rates were determined for all cultures treated with 0–2 μg mL^-1^ of fusidic acid. The final OD values for cultures exposed to fosfomycin over the same concentration range were higher than that of the antibiotic-free control, with the maximum OD observed in the presence of 1 μg mL^-1^. This result indicates that, in contrast to the duration of the lag phase, for some antibiotics neither the maximum specific growth rate nor the final OD value is a meaningful predictor of dose-dependent inhibitory effects on microbial growth.

### STRAIN IDENTIFICATION AND FURTHER CHARACTERIZATION

In addition to the 16S rRNA-based phylogenetic analysis of the isolate, which confirmed the identification of *E. faecium* strain UKI-MB07, the strain was also identified as *E. faecium* using the Rapid ID 32 STREP system and VITEK-MS. Results of additional *in vitro* susceptibility testing (microdilution technique according to ISO 20776-1 as well as Etest) confirmed the MIC values obtained with the MICRONAUT-S MRSA/IFSG GP4 microdilution plates, and showed either identical MIC values or those within a range of one to four dilution steps. The *E. faecium* strain was susceptible to ampicillin and thus representing an exceptional phenotype (Leclercq et al., 2013).

### EFFECT OF COMBINED Ag(0) NANOPARTICLE/ANTIBIOTIC TREATMENT ON *ENTEROCOCCUS* GROWTH DYNAMICS

**FIGURE 2:**
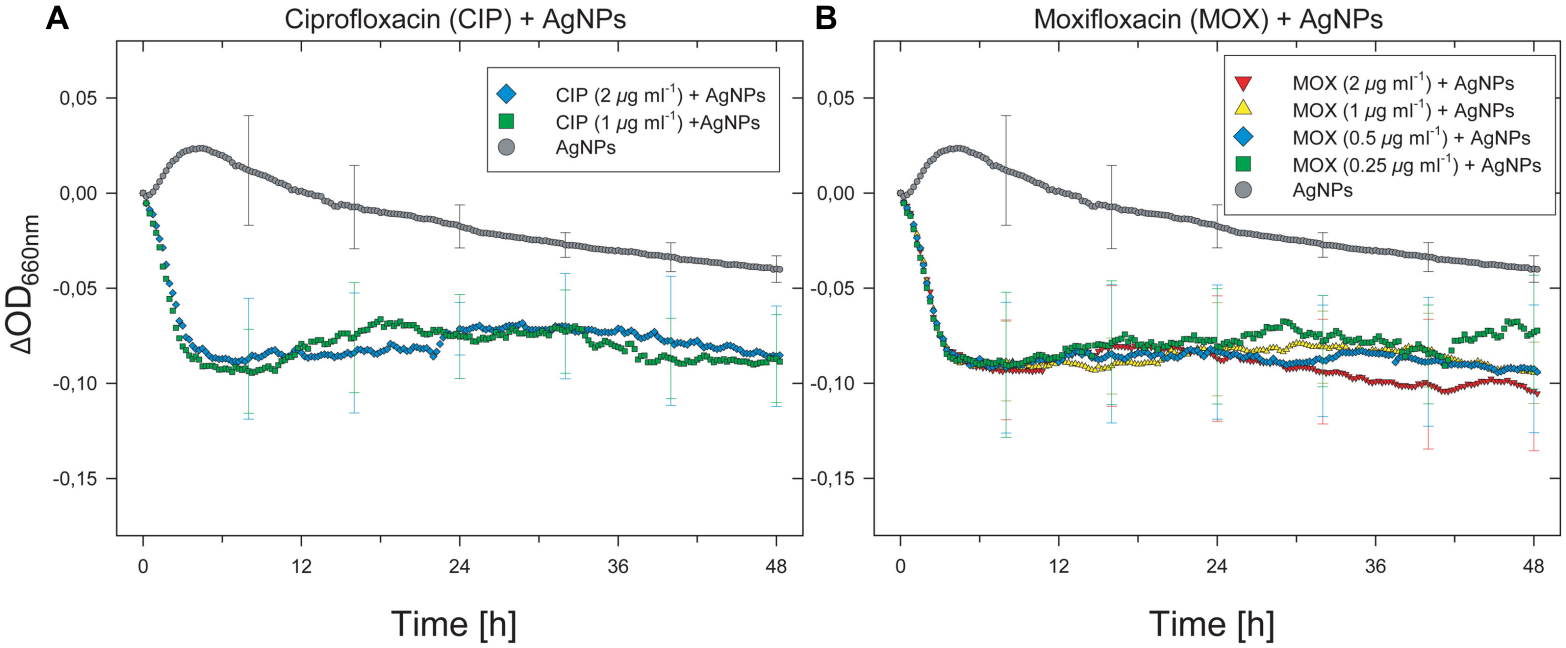
**Inhibition of growth of the *E. faecium* isolate by simultaneous treatment with antibiotics and silver nanoparticles**. Results for only ciprofloxacin and moxifloxacin are shown, but inhibitory effects were observed with all antibiotics tested. For visual clarity, SD (*n* = 3, error bars) are only presented for selected time points.

The monochromator-based instrument used in this study enables adjustment of the optical measurement settings in the presence of dispersed nanoparticles. Metal nanoparticles often exhibit strong background signals, which can affect absorbance measurements due to nanoparticle-specific (e.g., metal speciation and particle size distribution) and concentration-dependent properties. To examine putative synergism between antibiotics and metal nanoparticles, we compared the growth characteristics of the *Enterococcus* isolate in the presence of antibiotics or nanoparticles alone with its growth in the presence of both antibiotics and Ag(0) nanoparticles. Although growth was only partially inhibited by antibiotics alone at the tested concentrations (see **Figure 1**), the organism did not grow in the presence of both antibiotics and nanoparticles. The inhibitory effects of combined antibiotic/Ag(0) nanoparticle treatments involving ciprofloxacin and moxifloxacin are shown in **Figures 2A,B**.

### INTRACELLULAR ACCUMULATION OF REACTIVE OXYGEN SPECIES

**FIGURE 3:**
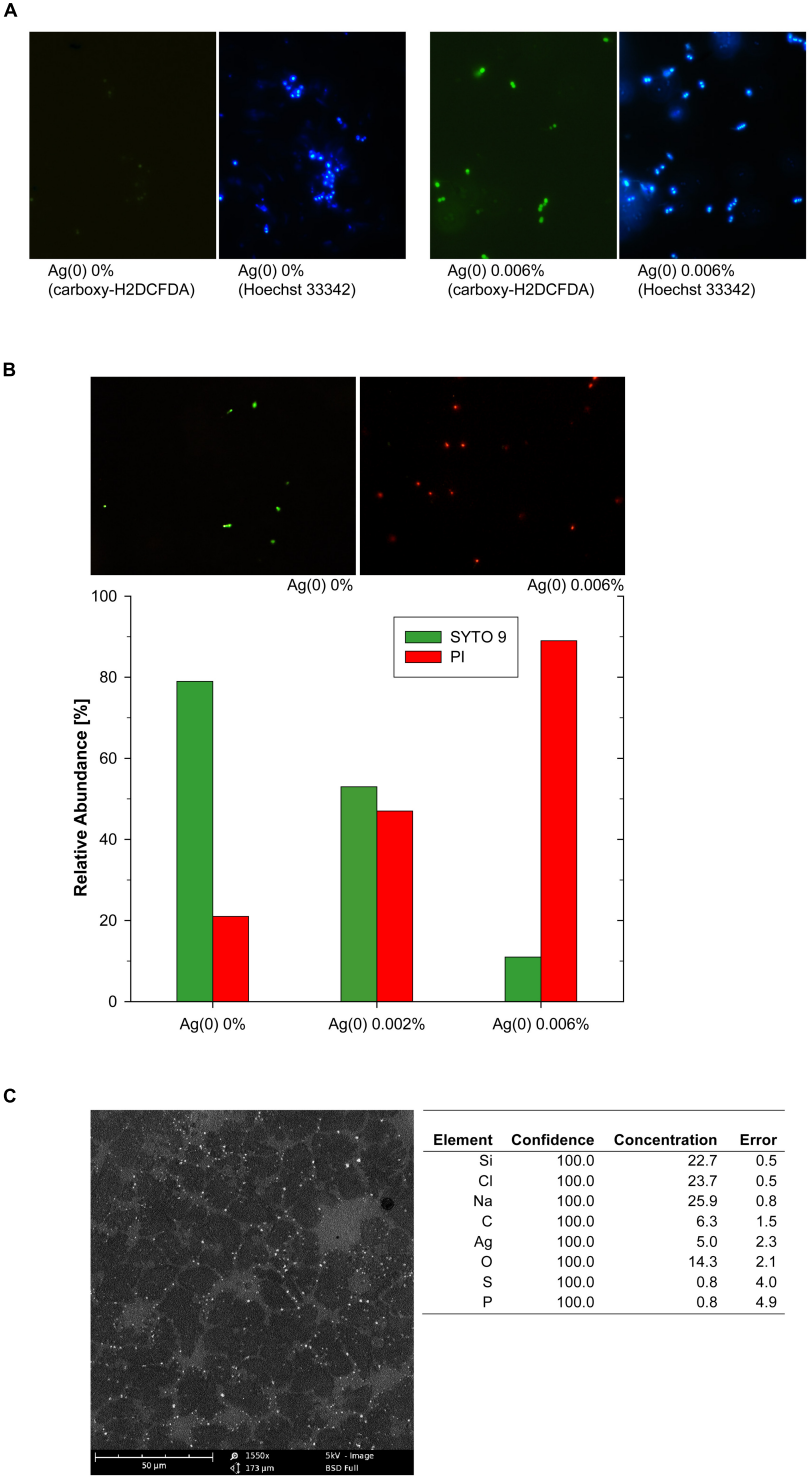
**Silver-nanoparticle mediated ROS accumulation in *Enterococcus* cells, shown by green fluorescence signals after carboxy-H_**2**_DCFDA staining, identical sections after counterstaining with Hoechst 33342 **(A)**, and assessment of cell membrane integrity at different Ag(0) nanoparticle concentrations **(B)**.** Representative images in **(B)** show SYTO 9 stained (green) *Enterococcus* cells with intact cell membranes and propidium iodide (red) stained cells with compromised cell membranes. SEM-EDX image of Ag(0) nanoparticles (0.006%, w/v) attached to cell debris in *Enterococcus* sp. cultures **(C)**.

The accumulation of intracellular ROS was assessed with a carboxy-H_2_DCFDA-based assay and accompanying examination by fluorescence microscopy. The exposure to Ag(0) nanoparticles led to intracellular accumulation of ROS as shown by strong green fluorescence in samples derived from 0.006% Ag(0) nanoparticle treatments (**Figure 3A**, right panels). Accumulation of ROS in *Enterococcus* cells did occur to a much lesser extent in the absence of Ag(0), as indicated by the presence of only weak fluorescence signals in Ag(0)-free control incubations (**Figure 3A**, left panels).

### CELL MEMBRANE INTEGRITY

In the absence of nanoscale Ag(0) particles, cell membrane disruption was only observed in about 20% of the *Enterococcus* cells after 48 h (**Figure 3B**). The exposure to Ag(0) nanoparticles led to cell membrane disruption of *Enterococcus* cells (**Figure 3B,C**), and the effects increased with increasing Ag(0) nanoparticle concentration (**Figure 3B**). As indicated by the predominance of red fluorescence signals after combined SYTO 9/PI staining, about 90% of the cells were considered as dead bacteria with damaged cell membranes in samples derived from 0.006% Ag(0) nanoparticle treatments (**Figure 3B**).

## DISCUSSION

A cultivation-based assay was employed to analyze the dynamic effects of various antibiotics on the growth of an *E. faecium* clinical isolate. Conventional testing of antibiotic susceptibility typically involves disk diffusion methods, antimicrobial gradient diffusion techniques, and broth (micro)dilution tests (Jorgensen and Ferraro, 2009). In accordance with the current reference methods (e.g., Clinical and Laboratory Standards Institute [CLSI], 2012, 2013), most recently published studies have reported antibiograms that are based on endpoint growth determination or analyses at discrete time points. However, on-line and real-time analysis of antibiotics susceptibility will provide by far more information for an optimized antibiotics treatment than static acquisition of single data points.

In our study, low concentrations of some antibiotics resulted in only partial inhibition of the growth of the *E. faecium* clinical isolate, as demonstrated by the extended lag phases, which would not have been observed in some cases if we had followed the recommended 16–20-h test duration. Importantly, different MICs were determined for some antibiotics after 20 and 48 h of susceptibility testing, including several “novel” and “reserve” antibiotics, such as daptomycin and vancomycin. This result underscores the importance of utilizing extended incubation periods for specific test organisms and antibiotics.

After the extended lag phase, the activity of some antibiotics correlated with reduced culture OD in the plateau phase, but contrary to our expectations, exposure to fosfomycin, daptomycin, fusidic acid, cefoxitin, or gentamicin led to reversible growth inhibition, which could be compensated for by higher cell densities until the end of the experiment. Exposing *E. faecium* UKI-MB07 to fosfomycin or fusidic acid even resulted in higher maximum specific growth rates. It is immediately evident from these results that conventional off-line antibiotic susceptibility testing is insufficient for tracking temporal changes in microbial growth profiles. Furthermore, our findings clearly indicate that acquisition and comparison of antibiotic resistance profiles should include analyses of dynamic growth profiles. There is a lack of reliable data illustrating the number of cases of antibiotic treatment failure associated with inadequate testing methods or testing periods too short in duration, which may result in misinterpretation of an organism’s susceptibility. This can lead to prolonged and excessive inflammation of affected tissues due to various substances produced by colonizing bacteria (e.g., proteases, toxins, and proinflammatory molecules), resulting in subsequent spread of the infection and possibly sepsis.

Our observation of limited promotion of *Enterococcus* growth under conditions of moderate stress (i.e., stimulation of growth in the presence of sublethal concentrations of antibiotics) in comparison to antibiotic-free controls is perplexing. The reason for such an effect is not yet clear, and we have not proven whether this phenomenon occurs *in vivo*. If so, the consequences with respect to our understanding of the actions of antibiotics and their proper application would be far-reaching, as it is not possible to ensure that the concentration of an antibiotic will remain high enough in all body compartments and tissues to eliminate an infection over the standard course of treatment (Theuretzbacher, 2007; Czock et al., 2009; Nau et al., 2010). The establishment of such “host” microcompartments may facilitate subinhibitory levels of antibiotics in such sections and during specific periods of treatment when the concentration of an antibiotic may decline below a specific threshold. Such scenario could expand enrichment, survival and promotion of antibiotic-tolerant organisms by known selection mechanisms (Aminov, 2009; Davies and Davies, 2010) to effects that occur when antibiotics are present at low concentrations, such as persistence, SOS response, hypermutation, direct mutagenic effects, and changes for intrachromosomal recombination and horizontal gene transfer (Rodriguez-Rojas et al., 2013). In addition, as our data for growth promotion in pure cultures of strain UKI-MB07 could indicate (at low antibiotics concentrations compared to antibiotic-free controls), this might be accompanied or accelerated by further mechanisms.

Beneficial (hormetic) effects associated with low concentrations of antibiotics were described early in the antibiotics era, and the ecological significance of these effects has been discussed previously (Miller et al., 1945; Davies et al., 2006; Linares et al., 2006; Martinez et al., 2009). Our results imply that low concentrations of antibiotics not only lead to selective enrichment of tolerant bacteria by enabling them to outcompete less tolerant species (Andersson and Hughes, 2011) and the described effects at subinhibitory levels (Rodriguez-Rojas et al., 2013), but they can actually promote the growth of tolerant species itself. Treatment with antibiotics may thus directly promote the enrichment and maintenance of antibiotics tolerance in microbial populations, and thus also involving enhanced dissemination of multidrug resistance.

*Enteroccoccus* spp. are increasingly reported as causative agents of nosocomial infections, including urinary tract infections and endocarditis (European Antimicrobial Resistance Surveillance System [EARSS] Annual Report, 2008; Fisher and Phillips, 2009; Arias and Murray, 2012; Victorian Advisory Committee on Infection Control [VACIC], 2012). Acquired antibiotic resistance, including vancomycin resistance, has gained considerable notoriety (Uttley et al., 1988; Klare et al., 2003; Fisher and Phillips, 2009; Arias and Murray, 2012). In contrast to treatment with antibiotics alone, we also examined antibiotic/Ag(0)-nanoparticle combinations, and found that they completely inhibited the growth of the *Enterococcus* isolate, with clearly different dynamics compared with sole antibiotic or sole nanoparticle treatments. Among other toxic effects, Ag(0) nanoparticles (and released silver ions) interact with DNA, proteins, and other phosphorus- and sulfur-containing cell constituents and generate ROS. ROS can significantly disrupt multiple metabolic pathways, thus inhibiting the core physiological functions of the cell (Nel et al., 2006, 2009; Su et al., 2009; Marambio-Jones and Hoek, 2010). Additionally, silver nanoparticles can attach to the cell surface and alter the physical and chemical properties of the cell membrane and cell wall, and the resulting destabilization disrupts critical functions such as cell division, permeability, osmoregulation, electron transport, and respiration (Nel et al., 2009; Su et al., 2009; Marambio-Jones and Hoek, 2010). In our study, the exposure to Ag(0) nanoparticles led to the disruption of *Enterococcus* cell membranes in a dose-dependent manner. Furthermore, Ag(0) nanoparticle treatment induced oxidative stress in the *Enterococcus faecium* test organisms, as shown by increasing accumulation of intracellular ROS. Also in light of other studies (Nel et al., 2009; Su et al., 2009; Marambio-Jones and Hoek, 2010), this suggests that oxidative stress and the involved cellular response could be a common mechanism for the adverse effects caused by antimicrobial metal- or metal oxide nanoparticles. The effects of Ag(0) nanoparticles, including the production of ROS, are amplified through the release of silver ions over time in a dose-dependent– and particle-size–dependent manner (Nel et al., 2006; Carlson et al., 2008; Liu and Hurt, 2010; Marambio-Jones and Hoek, 2010).

The use of silver nanoparticles in medicine seems promising. For example, when applied to the surfaces of surgical instruments, implants, endotracheal tubes, catheters, or wound dressings, silver nanoparticles can reduce the bioburden in open wounds and act as a barrier against further infection (Senior et al., 2012). Future studies should aim to investigate the occasionally observed lowered susceptibility or even resistance to Ag and potential cross- or coresistance to metals and antibiotics (Silver, 2003; Stepanauskas et al., 2006; Chopra, 2007; Schacht et al., 2013).

## CONCLUSION

Determination of the degree of bacterial resistance to antibiotics is an important part of the management of infectious diseases. Based on our results, we recommend acquisition of growth kinetic data for further strains and antibiotics and assessment of their eligibility as meaningful predictors for antibiotics susceptibility. If clinical relevance is evaluated, incorporation of dynamic growth analysis in standardized methods for culture-based antibiotic susceptibility testing and extension of testing periods could be suggested to enable detection of delayed or enhanced growth in the presence of some antibiotics. Antibiotic-induced growth promotion of specific microorganisms at rates higher than in the absence of antibiotics can have profound consequences for treatment outcomes and underscores the need for a thorough examination of potential *in vivo* hormetic effects associated with subinhibitory antibiotic concentrations. Treatment using antimicrobial nanoparticles appears to have promising additive or synergistic effects; however, the potential role of metal nanoparticles in driving the rise and/or spread of bacterial antibiotic resistance must be ruled out through additional studies.

## Conflict of Interest Statement

The authors declare that the research was conducted in the absence of any commercial or financial relationships that could be construed as a potential conflict of interest.
